# A Universal Tool Interaction Force Estimation Approach for Robotic Tool Manipulation

**DOI:** 10.3390/s25216619

**Published:** 2025-10-28

**Authors:** Diyun Wen, Jiangtao Xiao, Yu Xie, Tao Luo, Jinhui Zhang, Wei Zhou

**Affiliations:** Pen-Tung Sah Institute of Micro-Nano Science and Technology, Xiamen University, Xiamen 361005, China; wendiyun@stu.xmu.edu.cn (D.W.); 19920211151542@stu.xmu.edu.cn (J.X.); luotao@xmu.edu.cn (T.L.); jinhuizhang@xmu.edu.cn (J.Z.); weizhou@xmu.edu.cn (W.Z.)

**Keywords:** interaction forces estimation, tool dynamics identification, wrist force/torque sensor, creep compensation, sensor zero-drift

## Abstract

The six-degree-of-freedom (6-DoF) interaction forces/torque of the tool-end play an important role in the robotic tool manipulation using a gripper, which are usually indirectly measured by a robot wrist force/torque sensor. However, the real-time decoupling of the tool’s inertial force remains a challenge when different tools and grasping postures are involved. This paper presents a universal tool-end interaction forces estimation approach, which is capable of handling diverse grippers and tools. Firstly, to address uncertainties from varying tools and grasping postures, an online-identifiable tool dynamics model was built based on the Newton–Euler approach for the integrated gripper–tool system. Sensor zero-drift caused by factors such as the tool weight and prolonged operation is incorporated into the dynamic model and identified online in real time, enabling a coarse estimation of the interaction forces. Secondly, a spiking neural network (SNN) is specially employed to compensate for uncertainties caused by the wrist sensor creep effect, since its temporal processing and event-driven characteristics match the time-varying creep effects introduced by tool changes. The proposed method is experimentally validated on a robotic arm with a gripper, and the results show that the root mean square errors of the estimated tool-end interaction forces are below 0.5 N with x, y, and z axes and 0.03 Nm with $\tau_{x}$, $\tau_{y}$, and $\tau_{z}$ axes, which has a comparable precision with the in situ measurement of the interaction forces at the tool-end. The proposed method is further applied to robotic scraper manipulation with impedance control, achieving the interaction forces feedback during compliant operation precisely and rapidly.

## 1. Introduction

Robotic tool manipulation capability is increasingly demanded in various application domains such as home assistance, industrial manufacturing, and minimally invasive surgery [1,2]. Particularly in robotic-assisted minimally invasive surgery, force-feedback-based manipulation control can prevent unintended tissue damage [3]. Dexterous tool manipulation requires the robot to flexibly grasp, use, and switch between different tools in-hand, similar to the way humans use pliers, screwdrivers, surgical scalpel, or scrapers [4,5].

The accurate measurement of interaction forces at the tool end is a critical prerequisite for robotic tool manipulation control [6]. Due to size constraints in practical applications, a common approach is to use a wrist-mounted six-axis force/torque sensor to indirectly measure external forces acting on the robot [7,8,9]. However, because of its mounting position, the sensor cannot directly measure the interaction forces at the tool end. Instead, it provides a coupled output that includes the inertial forces of both the gripper and the tool [10,11]. Therefore, it is essential to rapidly identify the inertial parameters (such as the mass, center of mass, and inertia tensor) of the tool and thereby accurately decouple the tool-end interaction force from the wrist force/torque sensor when the robot changes tools and their in-hand grasping postures.

Newton–Euler-based dynamic modeling combined with least squares parameter identification is widely applied to identify the tool inertial parameters and decouple the gravitational force, allowing for the accurate estimation of the net external force [12,13,14]. Enhancing the accuracy of tool inertial parameter identification has been the focus of extensive research efforts. Xie et al. [15] accounted for the effects of the sensor weight and the robot installation orientation in parameter identification and employed the least-squares method to estimate these parameters, thereby enhancing the identification accuracy. Zhang et al. [16] compensated for the sensor mounting offsets and installation angle deviations by leveraging the force sensor data from multiple robotic poses to identify the parameters, thereby enhancing the accuracy of static external force estimation. Farsoni et al. [17] incorporated physical consistency constraints into parameter identification to enhance the reliability of the identification parameters, employing nonlinear optimization to ensure compliance with rigid-body dynamics. Nadeau et al. [18] proposed a tool-mass discretization method to enable tool identification under trajectory constraints. This approach incorporates tool shape information to assist in parameter identification, thereby simplifying the model. Duan et al. [19] employed a self-developed wrist six-axis force/torque sensor with embedded linear and angular acceleration measurements, together with known payload mass and inertia, to compute and compensate inertial forces online. These studies demonstrate the effectiveness of Newton–Euler formulations in improving identification accuracy under static or structured trajectories. Beyond Newton–Euler-based dynamic modeling, several studies have also explored model-free approaches for estimating tool inertial forces during operation. Su et al. [20] used an artificial neural network (ANN) to identify the tool gravity by regressing the tool orientation against the sensor-projected force, then applied gravity decoupling and calibration to obtain the interaction force. Furthermore, Su in [21] proposed a model-free deep convolutional neural network (CNN) to identify the tool’s dynamic model and compensate the sensor measurements.

Another concern is that sensor-intrinsic errors—such as zero-drift, creep, and other uncertainties—can adversely affect accuracy during tool parameter identification and tool-end interaction force estimation [22,23,24,25]. Xue et al. [26] calculated and compensated the sensor’s zero-drift and gravitational components based on measurements under no external contact conditions. Yu et al. [27] utilized multi-pose sensor data and applied least-squares estimation combined with singular value decomposition (SVD) to identify the sensor’s zero-drift under constrained working conditions. Shin et al. [28] addressed sensor crosstalk (channel coupling in a six-axis force/torque sensor) and used gradient-based nonlinear optimization to simultaneously identify the sensor bias, tool gravity (center-of-mass torque), crosstalk parameters, and base inclination from a limited set of poses. Although these model-based methods can effectively remove steady-state biases, they are less capable of representing dynamic drift and coupling effects that evolve over time. Therefore, learning-based methods have been introduced as a complementary strategy, capable of capturing complex temporal correlations and achieving a higher precision in sensor error compensation. Yao et al. [29] applied a deep neural network (DNN) to predict the sensor zero-point—encompassing zero-drift, systematic errors, and self-weight effects—and compensated accordingly, reducing the tool-end interaction force error to below 1.5 N and the torque error to below 4.3 Nm. Liu et al. [30] proposed a two-stage identification framework for high-load, large-component collaboration scenarios, jointly identifying the static errors (base tilt and sensor zero-drift) and dynamic inertial parameters, and achieving accurate collision force perception via secondary zero-drift compensation during low-speed operation. Wu et al. [31] mitigated the force-signal creep induced by temperature drift by developing a time–temperature joint compensation model, with model parameters identified via particle swarm optimization (PSO), thereby enabling real-time temperature-drift compensation. Sun et al. [32] modeled the sensor output as a linear function of temperature, combined least-squares compensation with RBF-NN (Radial Basis Function Neural Network) nonlinear fitting, and employed a PSO-optimized LSSVM (Least Square Support vector machine) to achieve high-precision real-time temperature compensation with markedly reduced residuals. Gao et al. [33] addressed sensor creep by combining historical loading data with state classification and using an LSTM (Long Short-Term Memory) network for real-time creep prediction and compensation, significantly enhancing the measurement accuracy and stability.

In previous research, the tool was typically fixed at the end of the robotic arm and maintaining a constant relative pose. However, changing the tool types and grasping postures can induce varying levels of zero-drift and creep effects in wrist force/torque sensors. Previous methods did not account for these factors and were not validated under such conditions.

In this work, in addition to tool inertial force identification, we implemented a two-stage strategy for wrist sensor compensation. First, as the zero-drift errors caused by tool gravity and grasping variations change with the tool, we treated them as tool-induced characteristics and identified them during tool parameter identification. For minor uncertain errors with temporal characteristics, such as creep and temperature drift, we employed a Spiking Neural Network (SNN) for compensation, leveraging its advantages in processing time-series signals and event-driven capabilities. Noting that creep error is also tool-related, we created a training dataset incorporating payload information, enabling it to accurately identify creep errors that exhibit both temporal effects and tool-specific characteristics.

The contributions of this paper are as follows:

1. A high-precision and rapid tool-end force estimation approach is proposed for robotic tool manipulation, especially when the robot changes tools or their grasping postures during a manipulation task.
2. A two-stage wrist sensor compensation strategy is presented to achieve high accuracy in tool-end force estimation with a root-mean-square error below 0.5 N, through zero-drift error identification and creep error SNN-based compensation.
3. The proposed tool-end interaction force estimation method is implemented in a robotic manipulation controller and verified to be precise and rapid in a robotic adhesive scraping manipulation task.

The remainder of this paper is as follows: Section 2 provides a detailed description of the proposed method for identifying tool inertial parameters and presents the SNN-based compensation of sensor uncertainty errors. Section 3 shows the experiments and verifies the proposed method. In Section 4, we have conducted an analysis and discussion. Finally, Section 5 concludes this paper and suggests the directions for future work.

## 2. Methodology

### 2.1. Force Decoupling

**Figure 1:**
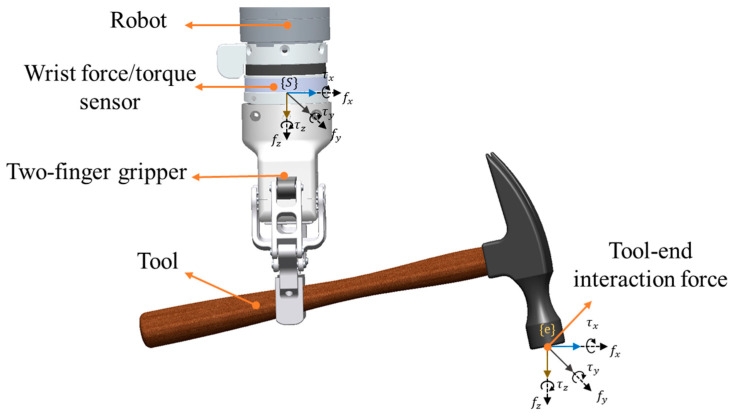
The installation of the wrist force/torque sensor and tool-end interaction forces in robotic tool manipulation.

In robotic tool manipulation, the wrist force/torque sensor is installed between the robot’s end effector and gripper, show in Figure 1. The gripper grip different tools to perform manipulation tasks. The output of the wrist force/torque sensor includes the following:

(1) Fs=Fgs+F0+Fu+Fe,

where $F_{s}=[f_{sx},f_{sy},f_{sz},\tau_{sx},\tau_{sy},\tau_{sz}{]}^{T}$ is the raw measurement values from the wrist force/torque sensor, Fgs=[fgx,fgy,fgz,τgx,τgy,τgz] is the inertial force and torque caused by the gripper-tool, $F_{0}=[f_{0x},f_{0y},f_{0z},\tau_{0x},\tau_{0y},\tau_{0z}{]}^{T}$ is the zero-drift of the sensor, $F_{u}=[f_{ux},f_{uy},f_{uz},\tau_{ux},\tau_{uy},\tau_{uz}{]}^{T}$ is the uncertainties caused by wrist sensor creep effect, and $F_{e}=[f_{ex},f_{ey},f_{ez},\tau_{ex},\tau_{ey},\tau_{ez}{]}^{T}$ is the tool-end interaction force between the tool and the environment during manipulation.

### 2.2. Gripper–Tool Dynamic Parameter Identification

Based on Newton–Euler equations, the dynamic model of the gripper–tool assembly is established with respect to the robot end-effector coordinate frame {S}.

(2) fgs=mas−mgs+αs×mcs+ωs×ωs×mcsτgs=Isαs+ωs×Isωs+mcs×as−mcs×as

where fgs and τgs are the inertial force and torque caused by the gripper–tool on the sensor, m denotes the mass of gripper–tool, as denotes the linear acceleration vector, αs denotes the angular acceleration vector, ωs represents the angular velocity vector, gs represents the gravity vector, cs denotes the position of the gripper–tool’s center of mass, and Is represents the inertia tensor. All these parameters are described relative to the sensor coordinate system, denoted as {S}, specifically,

(3) Is=IxxIxyIxzIxyIyyIyzIxzIyzIzz

Given with Equation (2), the observation matrix A is then separated as follows:

(4) Fgs=fgτg=A(as,αs,ωs,gs)φs,

where A(as,αs,ωs,gs)∈R6×10 and the expressions of A(as,αs,ωs,gs) is

(5) A(as,αs,ωs,gs)=ax−gx−ωy2−ωz2ωxωy−αzωxωz+αy000000ay−gyωxωy+αz−ωx2−ωz2ωyωz−αx000000az−gzωxωz−αyωyωz+αx−ωy2−ωx200000000az−gz−ay+gyαx−ωxωz+αyωxωy+αz−ωyωzωy2−ωz2ωyωz0−az+gz0ax−gxωxωzωyωz+αxωz2−ωx2αy−ωxωy+αz−ωxωz0ay−gy−ax+gx0−ωxωyωx2−ωy2−ωyωz+αxωxωyωxωz+αyαz,

φs is the parameters’ vector to be identified,

(6) φs=[m,mcsx,mcsy,mcsz,Ixx,Ixy,Ixz,Iyy,Iyz,Izz]T.

Inspired by reference [33], we recognized that varying tool qualities and distinct force application modes can induce differing initial zero-drift effects in sensors. Consequently, in this section, the zero-drift of the sensor $F_{0}$ is treated as an additional parameter and then added to the inertial parameters of the tool for unified identification. The expanded equation is now represented as follows:

(7) F=Aextφexts,

where $F=F_{0}+F_{g}$ is the non-interactive force portion of the sensor measurement, $A_{ext}=[E_{6\times 6}A]$ denotes extended regression matrix, and φsext=[f0x,f0y,f0z,τ0x,τ0y,τ0z,φs]T is the expanded parameter vector.

The least-squares method (LSM) is characterized by high computational efficiency, accuracy, and real-time capability, making it suitable for real-time tool dynamic parameter identification. Therefore, the identified parameters are as follows:

(8) φexts=(AextTAext)−1AextTF.

To optimize the tool’s dynamic behavior and minimize identification errors, the formulation of an appropriate excitation trajectory is crucial. Trajectories generated using Fourier series and polynomials are periodic, ensuring smooth initiation and termination, minimal jitter, and reduced data noise [34]. The trajectory expression is articulated as follows:

(9) $$\theta_{i}\left(t\right)=\sum_{k=1}^{N}\left[\frac{a_{i,k}}{\omega_{f}k}\sin\left(\omega_{f}kt\right)-\frac{b_{i,k}}{\omega_{f}k}\cos\left(\omega_{f}kt\right)\right]+\sum_{i=0}^{5}c_{i,m}(t-\left\lfloor \frac{t}{T}\right\rfloor T{)}^{m},$$

where $\theta_{i}\left(t\right)$ represents the angle of joint *i* of the robotic arm, N is the number of harmonics in the Fourier series trajectory, k is the harmonic index, $\omega_{f}$ is the fundamental frequency, $a_{i,k}$ and $b_{i,k}$ are the coefficients of the sine and cosine components of the Fourier series, $c_{i,m}$ represents the coefficient of the m-th term in the fifth-degree polynomial, t denotes the trajectory running time, $T\;=\;2\pi /\omega_{f}$ denotes trajectory period, and ⌊ ⌋ denotes the floor function. The parameters $a_{i,k}$, $b_{i,k}$, and $c_{i,k}$ are the optimization variables.

To minimize the impact of data errors on identification accuracy, it is essential to reduce the condition number $k(\tilde{A})$ of the regression matrix and optimize the coefficients in the excitation trajectory within the specified constraints. The constraints are as follows:

(10) $$\left\{\begin{array}{l} \min(k(\tilde{A}))\\ \theta_{i,min}\leq \left|\theta_{i}(t)\right|\leq \theta_{i,max}\\ \left|{\dot{\theta }}_{i}\left(t\right)\right|\leq {\dot{\theta }}_{i,max}\\ \left|{\ddot{\theta }}_{i}\left(t\right)\right|\leq {\ddot{\theta }}_{i,max}\\ \theta_{i}\left(t_{0}\right)=\theta_{i}\left(t_{f}\right)\\ {\dot{\theta }}_{i}\left(t_{0}\right)={\dot{\theta }}_{i}\left(t_{f}\right)=0\\ {\ddot{\theta }}_{i}\left(t_{0}\right)={\ddot{\theta }}_{i}\left(t_{f}\right)=0\\ w(\theta \left(t\right))\subset W_{0} \end{array}\right.,$$

where $\theta_{i,min}$ and $\theta_{i,max}$ are the angle limits of joint i, where ${\dot{\theta }}_{i,max}$ and ${\ddot{\theta }}_{i,max}$ represent the preset maximum joint angular velocity and angular acceleration, respectively; $t_{0}$ and $t_{f}$ are the initial and ending moments of the excitation trajectory, respectively; $w(\theta \left(t\right))$ represents the Cartesian space position of the robotic arm; and $W_{0}$ represents the movable space of the robotic arm.

The optimal excitation trajectory is then obtained by iterative optimization of the trajectory using MATLAB Optimization Toolbox (MATLAB R2023a, Optimization Toolbox Version 9.4), where $\omega_{f}\;=\;0.2\pi \;\mathrm{Hz}$, N=5, and *i* takes value from 3 to 6 to decrease the required working space and ensure the condition number meets identification requirements.

Control the robotic arm to follow the excitation trajectory without contact, that is, $F_{e}=0$. In this scenario, the measured values from the sensor are denoted as $F_{s}=F+F_{u}$, and n sets of sensor data and motion data as, αs, ωs, and gs are calculated with Jacobi. Substitute them into Equation (8); then, the extended model parameters can be obtained as follows:

(11) φexts=(A~extTA~ext)−1A~extTF~,

where $\tilde{F}=[F_{1},\;F_{2},\ldots ,F_{n}{]}^{T}$ represents the 6n dimensional column vector composed of *n* groups of sensor data, and ${\tilde{A}}_{ext}=[A_{{ext}_{1}},\;A_{{ext}_{2}},\ldots ,A_{{ext}_{n}}{]}^{T}$ is the 6*n* × 16 dimensional observation matrix composed of n groups of motion data.

By substituting the identified parameters into Equation (11) and using Equations (4) and (7) to decouple the sensor measurements, the interactive force at the tool-end of the robot arm leads to

(12) Fe+Fu=Fs−F=Fs−A~extφexts

### 2.3. Sensor Uncertain Error Compensation

After accounting for the inertial forces of the gripper–tool assembly and the sensor’s zero-drift error, the readings from the wrist force/torque sensor still include uncertain errors $F_{u}$ due to sensor creep effects, as expressed in Equations (1) and (12). A novel deep neural network, a spiking neural network (SNN) [35], is employed to identify the uncertain force $F_{u}$. In contrast to traditional neural networks such as MLP (Multilayer Perceptron), CNN, and LSTM, SNNs possess inherent temporal dynamics and sparse computation capabilities, allowing them to effectively process time-varying signals such as time-varying sensor creep and temperature drift. Furthermore, the event-driven nature of SNNs contributes to high computational efficiency, and, together with their low power consumption, makes them well-suited for robotic tool manipulation applications. Leveraging these advantages, this study adopts SNNs for error compensation to enhance both the accuracy and responsiveness of tool-end interaction force estimation during dynamic manipulation tasks.

**Figure 2:**
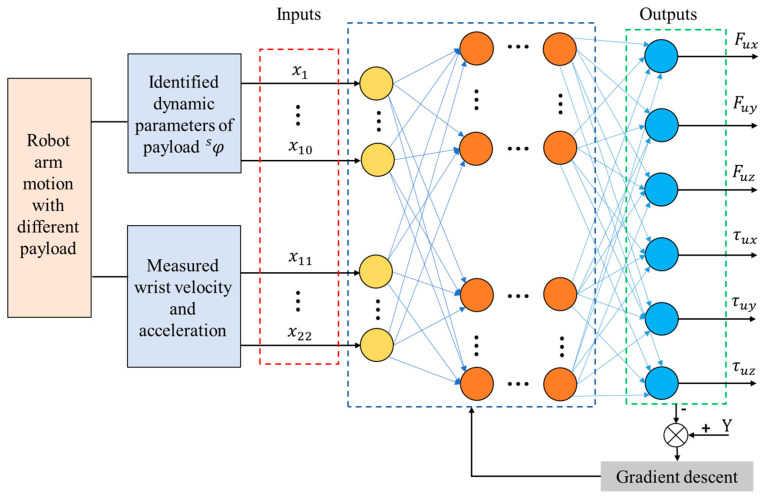
Sensor uncertainty error estimation with Spiking Neural Network.

The proposed SNN-based uncertainty errors estimation method is shown in Figure 2. The output of the training model is defined as $F_{u}$, which is calculated from the reading of the wrist sensor and the identified force F in Equation (7). The inputs include the 10 dynamic parameters φs of the payload and the measured wrist 6-dimensional velocity and acceleration vector, respectively. The optimizer employs the Adam algorithm as implemented in PyTorch (Version 2.2.1), with a learning rate of 0.001. The number of hidden layer neurons is set to 128, and the output layer dimension corresponds to the output parameter dimensions. The membrane potential decay rate β and the threshold $V_{thr}$ are randomly initialized. The iteration count is set to 50,000, along with the optimizer and loss function. The regression model is trained iteratively, where forward propagation computes the error loss and updates the model weights W and bias b; backward propagation adjusts the membrane potential decay rate β and the threshold $V_{thr}$.

The loss function for training is set as follows:

(13) $$fit=\left|F_{u}-F_{pu}\right|,$$

where $F_{pu}$ denotes the output of the Spiking Neural Network model, which is the predicted value of the uncertainty error.

To increase the sample size and fully reflect the creep characteristics of the sensor under different motion states, a 60-second robot arm motion trajectory was commanded as the sample trajectory for data collection. Four sets of payloads (0.89 kg, 1.81 kg, 2.60 kg, and 3.51 kg) were attached to the robot. The wrist velocity, acceleration, and wrist force/torque data were collected at a frequency of 100 Hz, resulting in a total of 240,000 datasets, all of which were filtered. These data were aggregated into a training dataset incorporating temporal information and fed into the SNN for training.

To mitigate its effect on model accuracy, we employ L1 regularization to minimize model complexity and K-fold cross-validation to assess the model’s performance in generalizing to noisy data. We use L1 loss (absolute error) and the root mean square (RMS) of the difference as evaluation metrics and calculate the average of the K-fold cross-validations to determine the final evaluation metric. During cross-validation, we select the model parameters that perform best overall on the validation set as the final regression model. This method significantly improves the robustness of the model and effectively avoids overfitting by utilizing all the data.

## 3. Experimental Verification

### 3.1. Experimental Setups

**Figure 3:**
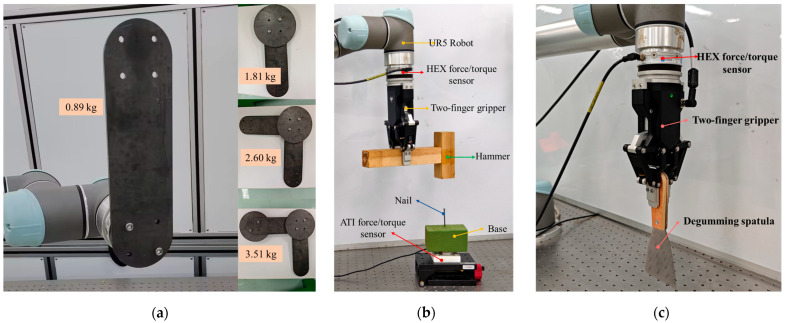
Experimental setups: (**a**) tool dynamic parameter identification and verification, (**b**) tool-end interaction force validation in a robotic nail-hammering task, and (**c**) robotic glue-scooping manipulation in which the estimated tool-end interaction force is used as feedback to the impedance controller.

To validate the effectiveness of the proposed tool force estimation method, a series of experiments were designed and conducted. The experimental setup, as shown in Figure 3, consists of a robotic arm (UR5, Universal Robots, Odense, Denmark), a six-axis force/torque sensor (HEX-E, OnRobot, Odense, Denmark), and a two-finger gripper (AG95, Dahuan Robotics, Suzhou, China).

Figure 3a shows the standard payloads of different masses, which were used for tool inertial parameters identification experiments. Figure 3b shows the robotic nail-hammering experiment design, which was employed to evaluate the effectiveness of the proposed tool-end interaction force estimation method during a tool manipulation task. A high-precision force sensor (Nano43, ATI Industrial Automation, Apex, NC, USA) with a sensing resolution of 0.008 N was used to measure the actual tool-end interaction force, that is, the hammering force applied to the nail. And Figure 3c presents a glue scooping manipulation task, in which the estimated tool-end interaction force serves as feedback to the impedance controller. Each experiment, including the hammering and adhesive-scraping tasks, was repeated at least three times under identical conditions to verify the consistency. The results from one representative trial are presented in the figures for clarity.

### 3.2. Identification of Tool Inertial Parameters

To verify the effectiveness and accuracy of the proposed tool dynamic parameters identification method, four sets of standard payloads (0.89 kg, 1.81 kg, 2.60 kg, and 3.51 kg) were used by running the excitation trajectory. These parameters are used to calculate the inertial force of the tool. Table 1 displays the identified and theoretical values of the ten dynamic parameters for the four standard loads. The identification errors for the load mass parameters are 6.7%, 2.8%, 2.3%, and 2.0%, respectively. The average identification errors for the three center-of-mass coordinate parameters of the loads are 8.1%, 4.3%, 7.2%, and 5.3%, respectively. It should be noted that these center-of-mass identification errors also include installation and the geometric alignment errors of the standard payloads, meaning that the true identification uncertainty is smaller than the reported theoretical values. The average time required for parameter identification across four standard payloads was 5.78 s, demonstrating high real-time performance. These results demonstrate that the method employed in this study is time-efficient and highly accurate with minimal error.

It is observed that some theoretical values are 0 in Table 1, while their identified values are not. This is mainly due to the manufacturing errors of the homemade payloads and alignment error caused by mounting. Moreover, as the values of the inertia parameters of the payload are relatively small and primarily driven by acceleration, and considering the speed requirements for robotic tool operations, the components caused by the inertia parameters are generally minor in Equation (2). Nevertheless, the uncertainty introduced by the identified inertial terms may still propagate into the estimation of the gravitational and inertial force $F_{g}$, thereby affecting the overall accuracy of the estimated tool-end interaction force $F_{e}$. This effect remains limited under our low-speed experimental conditions but represents an inherent source of uncertainty within the identification process. This uncertainty could be further reduced through the optimization of the excitation trajectory design and the conditioning of the least-squares identification procedure.

### 3.3. Tool-End Interaction Force Estimation

The purpose of this experiment is to evaluate the accuracy of the proposed tool-end interaction force estimation method in a robotic nail-hammering scenario, as shown in Figure 3b. First, the robot grasps the wooden hammer without contacting the nail, indicating that the tool and its in-hand posture have been confirmed and that the tool-end interaction force $F_{e}$ is zero at this stage. We then proceed with gripper–tool dynamic parameter identification, inertial force decoupling, and SNN-based uncertainty error compensation. The total duration of the entire recognition and computation process is approximately 6.5 s.

**Figure 4:**
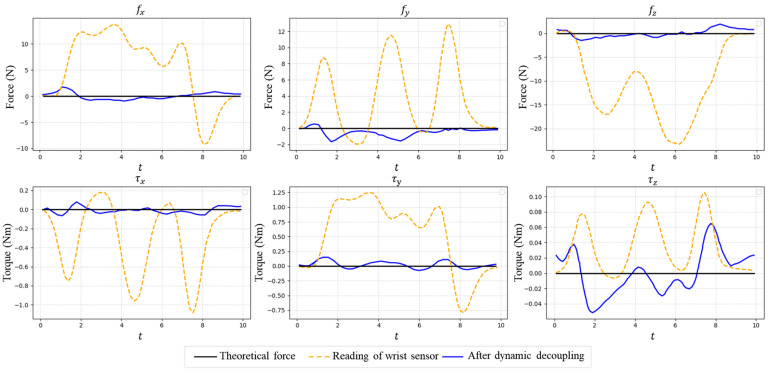
The reading of the wrist force/torque sensor and the estimated tool-end interaction force after inertial force decoupling.

**Figure 5:**
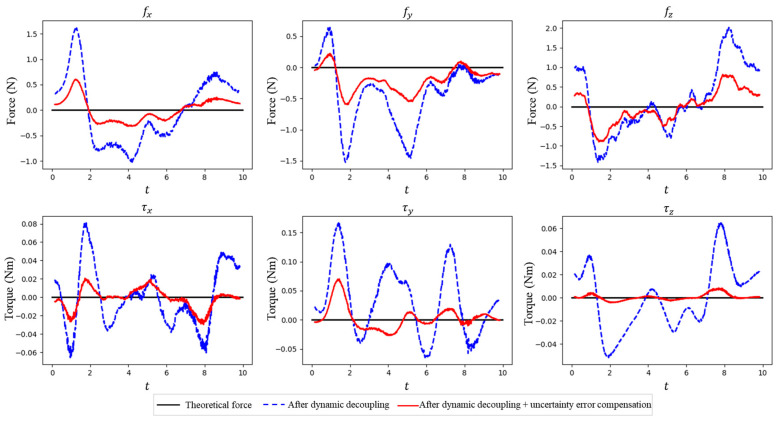
The estimated tool-end interaction force with and without uncertainty error compensation.

Figure 4 shows the reading of the wrist force/torque sensor and the estimated tool-end interaction force after inertial force decoupling. It can be seen that, after the decoupling of inertial forces, the tool-end interaction force approaches the actual value of zero; yet, a maximum estimation error of approximately 1.8 N still remains. Figure 5 demonstrates the estimated tool-end interaction force with and without uncertainty error compensation, which shows the maximum tool-end interaction force error can be further reduced to 0.8 N after uncertainty error compensation. We compare the average estimation error (RMSE) with the measurement accuracy of the wrist sensor in Table 2. These results partially show that our tool-end force estimation method achieves a precision comparable to the in situ measurement.

In order to verify the superiority of our method, we present the RMSE comparison of the six-axis force decoupling result between our method and other approaches when the robot is in a non-contact state (*F_e_* = 0) in Table 3, where the Double-LSM is the method proposed in the literature [16], which considers the sensor zero-drift and robot installation error. DL-LSM (Deep Learning—Least Squares Method) is the method proposed in the literature [28], and Bias with Non-linear Optimization is the method proposed in the literature [29], which considers not only the bias, center of mass, robot inclination, but also the crosstalk of the sensor. And the SNN-LSM is our method. In Table 3, our method achieves lower RMSE values on the $f_{x}$, $f_{y}$, and $f_{z}$ axes compared with other approaches. The RMSE values for the torque are slightly higher than other methods, particularly Ref. [29]. This is primarily because Ref. [29] estimated the torque using large sample sizes of torque-generating factors, including attitude-related zero points, mounting inclination angles, and the gravitational effects of tool. Therefore, only minor random noise remains in the contactless state, resulting in torque errors appearing very close to zero.

Given that these methods employ sensors with differing precision and measurement ranges, a simple comparison of force RMSE data post-identification may not accurately reflect the methodological superiority. Therefore, we calculated %FS values based on the RMSE values obtained from each method and the sensor parameters for a more reasonable comparison, as shown in Table 4. It is evident that our method exhibits a high accuracy on the force estimation of $f_{x}$, $f_{y}$, and $f_{z}$, while the gap with other methods on the torque is significantly narrowed.

Considering all six force/torque axes, our method demonstrates a superior overall accuracy in the six-axis force/torque estimation. This improvement results from our two-stage sensor error identification method. The method addresses the errors induced by varying tool types and grasping postures: the stable zero-drift is incorporated into the dynamic model for identification, while time-varying uncertainties from creep and temperature drift are compensated for by the SNN.

**Figure 6:**
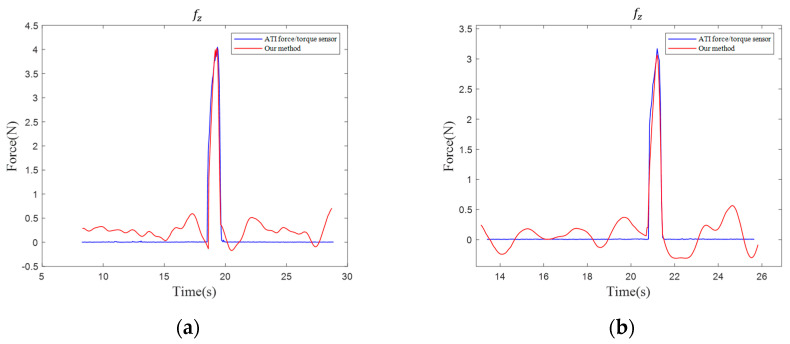
The force trajectories of the estimated hammer–nail interaction force and the actual measured values: (**a**) striking acceleration 50 mm/s^2^; and (**b**) striking acceleration 100 mm/s^2^.

We then allow the robot to strike the nail along the z-axis under two different acceleration conditions (50 mm/s^2^ and 100 mm/s^2^). Figure 6 shows the force trajectories of the estimated hammer–nail interaction force and the actual measured values from the ATI (ATI Industrial Automation, Apex, NC, USA) force sensor.

It is observed that the transient impact force generated upon contact between the hammer and the nail is effectively captured, manifested as a sharp increase in interaction force during the hammering task. For the two accelerations (50 mm/s^2^ and 100 mm/s^2^), the average errors in $f_{z}$ over the entire hammering process are only 0.32 N and 0.21 N, respectively. Both values are within the established accuracy envelope (z-axis RMSE = 0.49 N, 0.245%FS), corresponding to 0.160%FS and 0.105%FS on $f_{z}$. These results indicate that the proposed tool-end force estimation maintains its precision under dynamic impact conditions.

### 3.4. Tool-End Interaction Force for Manipulation Controller

**Figure 7:**
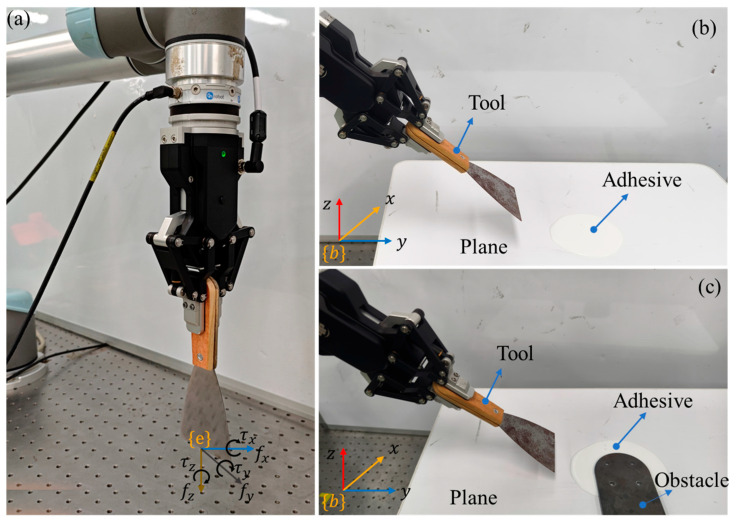
Robotic adhesive scraping manipulation task: (**a**) robot grasping a scraper; (**b**) scraping off the adhesive on a flat surface; and (**c**) scraping adhesive from an obstacled flat surface.

The purpose of this experiment is to evaluate the effectiveness and responsiveness of the proposed tool-end interaction force estimation method in a robotic adhesive scraping manipulation task. The ‘scrape off the adhesive’ task was chosen as the validation scenario because it requires the tool-end interaction force, as shown in Figure 7a, to be quickly and accurately estimated and serves as feedback to the manipulation impedance controller. The impedance control framework is detailed in [36].

**Figure 8:**
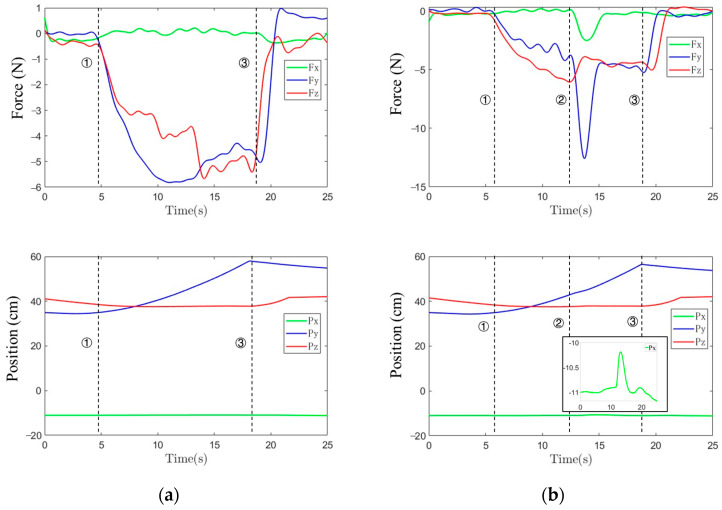
Regulated tool-end force and position trajectories during a robotic scraping manipulation control: (**a**) scraping off the adhesive from a flat surface; and (**b**) scraping off the adhesive from an obstacled flat surface.

During the adhesive scraping task, the robot grasps the scraper and moves in the positive y-direction of the world coordinate system {b}. The desired contact force of 5 N along the y-axis (motion direction) and z-axis (contact normal) are set as the input of the controller. Figure 8a,b show the regulated tool-end force and position trajectories during a robotic scraping manipulation control when the adhesive is on a flat surface and on an obstacled flat surface. It can be seen that, after establishing stable contact with a surface at the time point marked by dashed line 1, the controller is triggered and regulates the force values along the y-axis and z-axis near 5 N. In Figure 8b, the time point marked by dashed line 2 indicates the moment the scraper contacts the obstacle. This contact results in sudden changes in the force values along the x-axis and y-axis. The contact force in the x-axis direction causes the robot to generate a lateral displacement response within 0.02 s. This offset is approximately 0.9 cm, enabling the robot to successfully navigate around the obstacle. After navigating around the obstacle, the force values on the y- and z-axes quickly return to the pre-set contact force 5 N. For both Figure 8a,b, dashed line 3 indicates the end of the ‘scraping the adhesive’ task. Obviously, the estimated tool-end force can successfully be applied to a robotic scraping manipulation control.

## 4. Discussion

The above experiments verified that the proposed two-stage compensation method for zero-drift and creep error can precisely and rapidly decouple the tool-end interaction force from a wrist force/torque sensor, enabling robots to perform tool manipulation control. The tested four different payloads ranged from 0.89 kg to 3.51 kg, covering the typical mass range of most tools. As shown in Table 1, the identification error is largest when the payload is 0.89 kg, suggesting that the accuracy may degrade further for lighter tools due to the increased signal-to-noise ratio and quantization effects. Since the tool is grasped by a gripper, the identified payload includes both the gripper weight (1 kg) and the tool mass, making it incapable of identifying some light tools. In our nail-hammering and adhesive-scraping experiments, the wooden mallet and scraper employed weighed 246 g and 60 g, respectively. In robotic tool manipulation, consideration must be given not only to the tool selection but also to the permissible load of the robotic arm and the maximum load capacity of the gripper. As the maximum payload capacity of the gripper is 3 kg, and the robot itself is limited to 5 kg in the experiments, these constrains limit the performance verification beyond this range.

The method currently assumes a rigid tool model and fixed grasp configuration. Flexible or deformable tools could introduce dynamic coupling effects that are not captured by the rigid-body dynamics model, thereby reducing the estimation fidelity. Moreover, the SNN-based compensator was trained and executed on a single sensor configuration, and its long-term robustness to sensor aging or hardware degradation remains to be validated. The current method also neglects the possible crosstalk among different error components during dynamic identification. A further investigation of such crosstalk effects, from both theoretical and material perspectives, may further improve the accuracy of the interaction force estimation.

## 5. Conclusions

In this paper, we present an online identification method for the six-axis interaction forces at the end of any gripper and tool. This method combines the LSM and SNN to enhance the accuracy of tool-end interaction force identification through gripper–tool dynamic parameter identification, sensor zero-drift identification, and sensor uncertainty error compensation. The experimental results validated the accuracy of our proposed method for identifying the tool-end interaction forces, essentially achieving the in situ measurement of robotic tool-end interaction forces. We have also demonstrated that the force provided by this method exhibits a high precision and quick response within the impedance control framework. Future work will focus on extending the proposed online 6-DoF force/torque identification method to humanoid robots with multi-fingered hands. In addition, we will validate its performance in complex manipulation tasks involving multiple grasping modes and multi-contact configurations.

## Figures and Tables

**Table 1:** Standardized payload inertial parameter identification results.

	0.89 kg		1.81 kg		2.60 kg		3.51 kg	
	Identified	Theoretical	Identified	Theoretical	Identified	Theoretical	Identified	Theoretical
*m* (kg)	0.95	0.89	1.86	1.81	2.66	2.60	3.61	3.51
*c_x_* (mm)	−81.8	−89	−127.4	−130	−0.9	0	−132.4	−137
*c_y_* (mm)	−0.35	0	−0.95	0	−153.5	−156	0.66	0
*c_z_* (mm)	2.66	2.9	6.93	6.50	8.63	9.80	16.1	15
*I_xx_* (kg·cm^2^)	3.34	3.94	−72.25	−523.6	499.80	753.58	−123.3	−965.21
*I_xy_* (kg·cm^2^)	1.76	0	−130.56	0	19.08	0	−419.01	0
*I_xz_* (kg·cm^2^)	−2.58	−2.27	−11.47	−38.28	41.91	0	−140.8	−96.46
*I_yy_* (kg·cm^2^)	144.50	119.16	−517.74	−156.74	26.76	41.73	−882.71	−526.64
*I_yz_* (kg·cm^2^)	4.10	0	−39.29	0	43.04	−45.41	31.403	0
*I_zz_* (kg·cm^2^)	154.78	122.91	−486.99	−146.79	73.94	773.39	−804.65	−426.39
Identification time (s)	5.6		5.8		6.1		5.6	

**Table 2:** The RMSEs of the tool-end force after decoupling and after SNN compensation when the robot is in a non-contact state ($F_{e}=0$).

	Reading of Sensor	After Decoupling	After SNN Compensation	Resolution of Wrist Sensor
$f_{x}$(N)	8.41	0.75	0.27	0.2
$f_{y}$(N)	5.70	0.74	0.31	0.2
$f_{z}$(N)	12.84	1.02	0.49	0.8
$\tau_{x}$(N*m)	0.477	0.057	0.023	0.01
$\tau_{y}$(N*m)	0.791	0.081	0.028	0.01
$\tau_{z}$(N*m)	0.049	0.031	0.008	0.002

**Table 3:** Comparison of RMSE of six-axis force/torque error with other studies.

	$f_{x}$	$f_{y}$	$f_{z}$	$\tau_{x}$	$\tau_{y}$	$\tau_{z}$	Method
Ours	0.27	0.31	0.49	0.023	0.028	0.008	SNN-LSM
[16]	0.593	0.751	0.822	0.013	0.301	0.003	Double-LSM
[28]	0.693	1.069	1.285	0.095	0.110	0.022	Bias with Non-liner Optimization
[29]	0.377	0.583	0.629	0.0082	0.0138	0.003	DL-LSM

**Table 4:** Comparison of %FS of 6-axis force/torque error with other studies.

	$f_{x}$ (%FS)	$f_{y}$ (%FS)	$f_{z}$ (%FS)	$\tau_{x}$ (%FS)	$\tau_{y}$ (%FS)	$\tau_{z}$ (%FS)	Method	Sensor Resolution
Ours	0.135	0.155	0.245	0.230	0.280	0.123	SNN-LSM	HEX-E OnRobot ($f_{x,y}$ 0.2, $f_{z}$ 0.8, $\tau_{x,y}$ 0.01, $\tau_{z}$ 0.002)
[16]	0.473	0.571	0.328	0.218	0.428	0.049	Double-LSM	Omega190 ($f_{x,y}$ 0.375, $f_{z}$ 0.628, $\tau_{x,y}$ 0.05, $\tau_{z}$ 0.03)
[28]	0.347	0.535	0.643	0.633	0.733	0.147	Bias with Non-liner Optimization	AFT200 ($f_{x,y,z}$ 0.15, $\tau_{x,y,z}$ 0.015)
[29]	0.377	0.583	0.315	0.164	0.276	0.060	DL-LSM	Self-developed tandem force sensor ($f_{x,y}$ 0.003, $f_{z}$ 0.006, $\tau_{x,y,z}$ 0.00015)

## Data Availability

The original contributions presented in this study are included in the article. Further inquiries can be directed to the corresponding author.
